# Combating Enteroaggregative *Escherichia coli:* Dual antibacterial and antibiofilm effects of silver- and copper-1,10-phenanthroline-5,6-dione complexes

**DOI:** 10.3934/microbiol.2025036

**Published:** 2025-11-18

**Authors:** Caroline Gastaldi Guerrieri, Mariane Vedovatti Monfardini Sagrillo, Solange Alves Vinhas, Michael Devereux, Malachy McCann, Thaís Pereira de Mello, Liliana Cruz Spano, André Luis Souza dos Santos

**Affiliations:** 1 Department of Pathology, Health Sciences Center, Federal University of Espírito Santo, Vitória, Espírito Santo, Brazil; 2 Núcleo de Doenças Infecciosas, Health Sciences Center, Federal University of Espírito Santo, Vitória, Espírito Santo, Brazil; 3 Centre for Biomimetic and Therapeutic Research, Focas Research Institute, Technological University Dublin, Dublin, Ireland; 4 Chemistry Department, Maynooth University, Maynooth, Ireland; 5 General Microbiology Department, Institute of Microbiology Paulo de Góes, Federal University of Rio de Janeiro, Rio de Janeiro, Brazil

**Keywords:** EAEC, diarrhea, antimicrobial resistance, biofilm, coordination compounds, copper complex

## Abstract

Enteroaggregative *Escherichia coli* (EAEC) causes acute and persistent diarrhea. Its antimicrobial resistance and strong biofilm formation hinder treatment, highlighting the need for new therapies. This study evaluated the antimicrobial efficacy of 1,10-phenanthroline-5,6-dione (phendione) and its copper [Cu(phendione)_3_](ClO_4_)_2_.4H_2_O (Cu-phendione) and silver [Ag(phendione)_2_]ClO_4_ (Ag-phendione) complexes against planktonic and biofilm-forming EAEC cells. The minimum inhibitory concentration (MIC) and minimum bactericidal concentration (MBC) values were determined for planktonic cells of 35 clinical EAEC isolates, revealing potent antibacterial activity by all test compounds, with Cu-phendione showing the greatest efficacy, followed by Ag-phendione and phendione. Most combinations of Cu-phendione or Ag-phendione with either ampicillin or tetracycline exhibited additive effects through checkerboard assays, whereas time-kill experiments revealed synergistic interactions between the complexes and those classical antibacterial agents. Minimum biofilm inhibitory concentration (MBIC) analysis identified Cu-phendione as the most effective compound for disarticulating biofilm formation (geometric MBIC = 14.61 µM), followed by Ag-phendione (24.69 µM) and phendione (67.08 µM). Notably, Cu-phendione eradicated biofilms in 24 isolates (68.6%), while Ag-phendione and phendione achieved eradication in 11 (31.4%) and 6 (17.1%) isolates, respectively. Furthermore, the test complexes were able to disrupt established mature biofilms, as demonstrated by the crystal violet assay and scanning electron microscopy. In combination therapy, complete biofilm eradication was achieved in all clinical isolates tested when Cu-phendione was paired with cefoxitin, tobramycin, tetracycline, or ciprofloxacin. Collectively, phendione-derived complexes, particularly Cu-phendione, represent promising candidates for the treatment of EAEC infections in planktonic and biofilm-associated states.

## 1. Introduction

Enteroaggregative *Escherichia coli* (EAEC), one of the six recognized diarrheagenic *E. coli* pathotypes, is a leading cause of both acute and persistent diarrhea in multiple regions worldwide. The infection poses a particularly high risk of mortality among children aged 12–23 months [1]–[3]. Persistent EAEC infections have also been linked to long-term gastrointestinal dysfunction, contributing to malnutrition, stunted growth, and cognitive deficits, even in the absence of overt clinical symptoms [3],[4]. First-line antimicrobial therapies for EAEC-associated diarrhea typically include ampicillin, sulfamethoxazole/trimethoprim, tetracyclines, and quinolones, primarily due to their wide availability and low cost [3]–[5]. However, rising antimicrobial resistance among EAEC clinical isolates has been documented globally, compromising the efficacy of these conventional agents [3],[6]–[10]. Notably, recent findings from our group revealed that even initially susceptible EAEC clinical isolates acquired resistance to most tested antimicrobials when growing in biofilm form [10]. Furthermore, none of the evaluated antimicrobials (ampicillin, cefoxitin, cefotaxime, ceftriaxone, tobramycin, chloramphenicol, ciprofloxacin, tetracycline, and trimethoprim-sulfamethoxazole) were effective in eradicating EAEC biofilms [10].

The rising prevalence of bacterial resistance underscores the urgent need for novel therapeutic agents with effective antimicrobial properties. In this context, 1,10-phenanthroline-5,6-dione (phendione) and its metal-based complexes, [Cu(phendione)_3_](ClO_4_)_2_.4H_2_O (Cu-phendione) and [Ag(phendione)_2_]ClO_4_ (Ag-phendione), have demonstrated promising activity against clinically significant Gram-negative bacterial pathogens. In this context, these compounds have been shown to inhibit planktonic growth, prevent biofilm formation, and disrupt mature biofilms of *Pseudomonas aeruginosa*, *Acinetobacter baumannii*, and *Klebsiella pneumoniae* [11]–[13]. Notably, these metal-phendione complexes showed strong antimicrobial activity against carbapenemase-producing *K. pneumoniae* clinical isolates, markedly lowering the meropenem minimum inhibitory concentration (MIC) in some isolates to clinically achievable levels and thereby restoring antimicrobial susceptibility [13].

Phendione-derived metal complexes have been proposed to disrupt microbial metal homeostasis by interfering with the acquisition and bioavailability of essential metal ions required for critical cellular processes [14],[15]. These compounds can inhibit the activity of metal-dependent proteins, such as metalloproteases and transcription factors, thereby impairing key regulatory and enzymatic pathways. In addition, they have been shown to induce respiratory uncoupling, increase membrane permeability, and disrupt overall cellular homeostasis, all of which contribute to their antimicrobial activity [14],[15]. Beyond their antimicrobial properties, phendione-based complexes also exhibit notable anti-virulence effects against *P. aeruginosa*. Both Ag-phendione and Cu-phendione inhibit the activity of elastase B (LasB) by downregulating the *lasB* gene and effectively protect *Galleria mellonella* larvae from the toxic effects of purified LasB protein and bacterial secretions containing the enzyme [16]. Furthermore, these complexes can interact with *P. aeruginosa* DNA through hydrogen bonding, hydrophobic interactions, and electrostatic forces. Remarkably, Cu-phendione induces oxidative DNA damage, an effect that is significantly attenuated in the presence of free radical scavengers, underscoring the role of oxidative stress in its mechanism of action [17]. Interestingly, both Ag-phendione and Cu-phendione complexes have demonstrated favorable safety profiles in silico and in vitro, showing low cytotoxicity across various mammalian cell lines, including macrophages, fibroblasts, and lung epithelial cells. This biocompatibility has been further corroborated by *in vivo* studies in *G. mellonella* larvae, Swiss mice, and hamsters [14],[18]–[23]. Remarkably, treatment with these complexes does not significantly alter key renal and hepatic biomarkers in mice and hamsters, including urea and aspartate aminotransferase levels, reinforcing their potential for therapeutic application [14],[23].

Given the promising antimicrobial and antiviral properties of phendione-based complexes, we sought to evaluate their efficacy against clinical isolates of EAEC with distinct antimicrobial susceptibility profiles. Our investigation focused on both planktonic cells and biofilm-forming populations, aiming to assess the capacity of these complexes to inhibit growth and disrupt established biofilms. In addition, we explored the potential synergistic effects of combining Ag-phendione and Cu-phendione with conventional antimicrobials commonly used in the treatment of diarrheagenic infections. This combinatorial approach was designed to determine whether these complexes could enhance the activity of existing antimicrobials or restore their efficacy against resistant EAEC clinical isolates.

## 2. Materials and methods

### 2.1. Microorganisms

A total of 35 clinical EAEC isolates, recovered from pediatric patients with or without diarrhea and displaying diverse susceptibility profiles to conventional antimicrobial agents (Table 1) [10], were included in this study. In addition, the prototype strain EAEC 042 and two ATCC reference strains were analyzed: *E. coli* ATCC 25922, commonly used as a quality control strain, and *E. coli* ATCC 2469, selected for its resistance to carbapenems.

### 2.2. Test compounds

1,10-Phenanthroline-5,6-dione (phendione), [Cu(phendione)_3_](ClO_4_)_2_.4H_2_O (Cu-phendione), and [Ag(phendione)_2_]ClO_4_ (Ag-phendione) were synthesized according to the methodology previously described in the literature [24]. The compounds and their respective salts [Cu(ClO_4_)_2_.6H_2_O and AgClO_4_] were dissolved in dimethyl sulfoxide (DMSO) (Sigma-Aldrich, St. Louis, MO, USA) and stored at room temperature in the dark.

**Table 1.** Antimicrobial susceptibility profiles of EAEC clinical isolates and reference strains investigated in this study.

Bacterial isolates	Isolation source	ANTIMICROBIAL SUSCEPTIBILITY PROFILE								
		AMP	FOX	CTX	CRO	CIP	TOB	TET	CHL	SXT
6A	Diarrhea	S	S	S	S	S	S	S	S	S
39B	Diarrhea	S	S	S	S	S	S	S	S	S
89A	Feces	S	S	S	S	S	S	S	S	S
110C	Feces	S	S	S	S	S	S	S	S	S
139D	Diarrhea	S	S	S	S	S	S	S	S	S
146A	Feces	R	S	S	S	S	S	R	S	S
164A	Feces	S	S	S	S	S	S	R	S	S
Q010A	Feces	S	S	S	S	S	S	R	S	S
Q015B	Diarrhea	S	S	S	S	S	S	S	S	S
Q016B	Diarrhea	S	S	S	S	S	S	S	S	S
Q028B	Feces	S	S	S	S	S	S	S	S	S
Q110A	Feces	S	S	S	S	S	S	R	R	S
Q132C	Feces	R	S	S	S	S	S	S	S	S
Q158A	Feces	S	S	S	S	S	S	S	S	S
Q165A	Feces	S	S	S	S	S	S	S	S	S
Q193A	Feces	S	S	S	S	S	S	S	S	S
Q226A	Feces	S	S	S	S	S	S	S	S	S
Q255D	Feces	S	S	S	S	S	S	S	S	S
Q266B	Feces	S	S	S	S	S	S	S	S	S
Q268A	Feces	R	S	S	S	S	S	R	R	S
Q269D	Feces	S	S	S	S	S	S	I	R	S
Q287A	Feces	R	S	S	S	S	S	R	R	R
Q300A	Feces	S	S	S	S	S	S	S	S	S
Q304A	Diarrhea	R	S	S	S	S	S	R	R	R
Q320A	Feces	R	S	S	S	S	S	R	R	S
Q322A	Diarrhea	R	S	S	S	S	S	S	R	R
Q323A	Diarrhea	R	S	S	S	S	S	R	R	S
Q340A	Diarrhea	S	S	S	S	S	S	S	S	S
Q345F	Feces	R	S	S	S	S	S	R	R	S
Q370A	Feces	S	S	S	S	S	S	S	S	S
Q391B	Feces	S	S	S	S	S	S	S	S	S
Q426A	Feces	S	S	S	S	S	S	S	S	S
Q434A	Feces	R	S	S	S	S	S	R	R	S
Q436B	Feces	R	S	S	S	S	S	R	S	S
Q488B	Feces	S	S	S	S	S	S	S	S	S
EAEC 042	Diarrhea	S	S	S	S	S	S	S	S	R
ATCC 2469	Urine	R	R	R	R	R	R	R	ND	R
ATCC 25922	Clinical sample	S	S	S	S	S	S	S	S	S

Note: AMP: Ampicillin; FOX: Cefoxitin; CTX: Cefotaxime; CRO: Ceftriaxone; CIP: Ciprofloxacin; TOB: Tobramycin; TET: Tetracycline; CHL: Chloramphenicol; SXT: Trimethoprim-sulfamethoxazole; R: Resistant strains; I: Intermediate strains; S: Susceptible strains; ND: Non-determined.

### 2.3. Determination of minimum inhibitory concentration and minimum bactericidal concentration

The minimum inhibitory concentrations (MICs) of phendione, Ag-phendione, Cu-phendione, as well as the simple salts of silver and copper, were determined using the broth microdilution method following the CLSI M100-Ed33 guidelines [25]. Assays were performed in 96-well microplates containing cation-adjusted Mueller–Hinton broth (CAMHB) with compound concentrations ranging from 0.78 to 12.5 mg/L for phendione, Ag-phendione, and Cu-phendione, and from 3.125 to 100 mg/L for the simple salts [Cu(ClO_4_)_2_.6H_2_O and AgClO_4_]. The MIC was defined as the lowest concentration at which no visible turbidity was observed. In addition, the MIC values required to inhibit 50% (MIC_50_) and 100% (MIC_100_) of the tested bacterial isolates, as well as the geometric mean of the MICs (GM-MIC), were calculated. The minimum bactericidal concentration (MBC) was determined by spotting 10 µL from wells without visible bacterial growth onto nutrient agar, followed by incubation at 37 °C for 24 h. The MBC corresponded to the lowest concentration of each compound at which no bacterial growth was observed [26].

### 2.4. Checkerboard assay

As previously reported, tetracycline and ampicillin exhibited the highest resistance rates among the EAEC clinical isolates in our collection (Table 1) [10]. Based on this finding, 10 tetracycline-resistant and 8 ampicillin-resistant isolates were selected to assess the effects of these antimicrobials in combination with Cu-phendione and Ag-phendione. Combination assays were performed in 96-well microplates using the checkerboard method, with serial dilutions of the test compounds prepared by columns and antimicrobials by rows, as previously described [27]. Concentrations ranged from 1/16× to 2× the MIC for each antimicrobial and from 1/8× to 2× the MIC for the test compounds. The results were interpreted by calculating the fractional inhibitory concentration index (FICI) according to the formula: FICI = (MIC of drug A in combination / MIC of drug A alone) + (MIC of drug B in combination/MIC of drug B alone). The FICI values were classified as follows: Synergy (FICI ≤ 0.5), additivity (0.5 < FICI < 2.0), indifference (2.0 ≤ FICI < 4.0), and antagonism (FICI > 4.0) [27].

### 2.5. Time-kill assay

To confirm the effects of combining Cu-phendione and Ag-phendione with therapeutic antimicrobials (ampicillin and tetracycline), time-kill assays were performed using two EAEC isolates selected based on the checkerboard results. Test compounds and antimicrobials were prepared at 0.5× MIC for each isolate, both individually and in combination, in CAMHB. Bacterial suspensions (10^6^ CFU/mL) were added to the prepared solutions and incubated at 37 °C. Samples (20 µL) were collected at 0, 3, 6, 9, 12, and 24 h, subjected to 10-fold serial dilutions in 0.85% saline (w/v), plated on nutrient agar, and incubated at 37 °C for 24 h before colony counting. Synergy was defined as a ≥2 log_10_ CFU/mL reduction in the combination compared to the most active single agent at 24 h. Bacteriostatic and bactericidal activities were defined as <3 log_10_ and ≥3 log_10_ reductions in CFU/mL, respectively, relative to the initial inoculum.

### 2.6. Determination of minimum biofilm inhibitory concentration and minimum biofilm eradication concentration

The minimum biofilm inhibitory concentration (MBIC) of phendione, Cu-phendione, Ag-phendione, and the corresponding simple salts was determined using the peg-lid system (Calgary device) [28] under conditions standardized by Sheikh and co-workers [29]. Briefly, each EAEC isolate was grown in LB broth (Kasvi, Italy) for 24 h and adjusted to 1.5 × 10^8^ CFU/mL; 200 µL of this suspension was added to 5.8 mL of Dulbecco's modified Eagle's medium (DMEM) (Cultilab, São Paulo, Brazil) supplemented with 0.4% (w/v) glucose. From this mixture, 150 µL was transferred to polystyrene microplates fitted with peg lids (Thermo Scientific™ Nalgene™, USA) and incubated at 37 °C with shaking at 110 rpm for 18–24 h. Mature biofilms on the pegs were washed, transferred to microplates containing serial twofold dilutions of each test compound in CAMHB (Sigma-Aldrich, Missouri, USA) at concentrations ranging from 0.78 to 50 mg/L, and incubated for 18–24 h at 37 °C. Biofilms were then recovered by sonication into fresh CAMHB, and optical density was measured at 620 nm immediately (t = 0 h) and after 6 h of incubation (t = 6 h). Experiments were considered valid if the mean OD_620_ increase of the growth control after 6 h minus the initial OD_620_ was ≥0.05 for each strain [29]. MBIC was defined as the lowest compound concentration yielding ≤10% of this growth [(OD_620_ t = 6 h − OD_620_ t = 0 h) × 0.1]. The MBIC_50_ and MBIC_100_ values, as well as the geometric mean MBIC (GM-MBIC) for each test compound, were calculated. The minimum biofilm eradication concentration (MBEC) for phendione, Cu-phendione, and Ag-phendione was determined by subculturing 10 µL from wells showing no turbidity onto nutrient agar; the MBEC was defined as the lowest concentration at which no bacterial growth was observed.

### 2.7. Ultrastructural analysis of biofilms

The biofilm of the EAEC Q255D, a strong biofilm-forming isolate [10] selected for this analysis, was developed using the peg-lid system as described above and treated for 24 h with Cu-phendione and Ag-phendione at their respective MBICs determined in this study. Following treatment, pegs were washed with phosphate-buffered saline (PBS) and fixed with Karnovsky fixative (2.5% glutaraldehyde, 2% formaldehyde, 0.1 M cacodylate buffer). Pegs were then washed three times with 0.1 M cacodylate buffer, dehydrated through an ethanol gradient, and dried using CO_2_ critical point drying. Samples were mounted on SEM stubs, gold-coated using a Denton Vacuum Desk V Sputter Coater (Denton Vacuum, Moorestown, NJ), and imaged with a JEOL JSM-6610 LV scanning electron microscope (JEOL, Peabody, MA). In parallel, bacterial cell size was quantified by measuring 100 randomly selected cells per treatment using ImageJ.

### 2.8. Effects of test compounds on the disarticulation of mature biofilm

To evaluate the effect of Cu-phendione and Ag-phendione on the disruption of mature biofilms formed by the 35 EAEC clinical isolates, biofilms were prepared using the peg-lid system as described above. Pegs with established biofilms were transferred to microplates containing serial dilutions of the test compounds (0.78–25 µg/mL) in CAMHB and incubated for 18–24 h at 37 °C. After incubation, peg lids were washed with PBS to remove residual compounds and non-adherent cells, dried for 20 min at room temperature, and fixed in 75% ethanol (v/v) for 10 min. Following a second drying step, pegs were stained with 0.5% crystal violet (w/v) for 5 min at room temperature, washed with distilled water, and dried for 1 h. The biofilm-bound dye was solubilized in 95% ethanol for 2 min, and the peg lids were discarded. Biofilm biomass was quantified by measuring optical density at 570 nm. Untreated biofilms on peg lids served as controls.

### 2.9. Effects of test compounds on biofilm formation kinetics

To evaluate the ability of phendione-derived compounds to interfere with EAEC biofilm formation, the biofilm formation kinetics of five EAEC clinical strains (three strong and two weak biofilm formers), along with the prototype strain EAEC 042, were assessed in the presence and absence of Cu-phendione and Ag-phendione. Bacterial isolates were first grown on nutrient agar for 24 h, followed by cultivation in LB broth for another 24 h at 37 °C. Bacterial suspensions were then prepared at 5 × 10^6^ CFU/mL in DMEM supplemented with 0.4% (w/v) glucose and added to 96-well microplates. Cu-phendione and Ag-phendione were added at 0.5× MIC, and the plates were covered with peg lids and incubated at 37 °C with shaking at 110 rpm. Controls consisted of bacterial suspensions in DMEM without compounds. At 3, 6, and 24 h of incubation, peg lids were washed to remove non-adherent cells and stained with crystal violet, as previously described, to quantify total biofilm biomass at each time point.

### 2.10. Effects of test compounds in combination with therapeutic antimicrobials on biofilm inhibition and eradication

Biofilms were prepared as described above, and peg lids were transferred to microplates containing combinations of test compounds (Cu-phendione or Ag-phendione) with therapeutic antimicrobials, including those to which the EAEC isolates were susceptible (cefoxitin, tobramycin, and ciprofloxacin) and resistant (ampicillin and tetracycline), arranged in a checkerboard format. Four bacterial isolates were selected for each combination. Plates with peg lids were incubated for 18–24 h at 37 °C. After incubation, peg lids were washed and transferred to fresh microplates containing CAMHB, followed by sonication for 10 min at 40 kHz to recover biofilms. Optical density at 620 nm was measured immediately (t = 0 h) and after 6 h of incubation at 37 °C (t = 6 h). Growth controls consisted of each strain cultured in the absence of compounds and antimicrobials. Concentrations of combinations that inhibited biofilm growth were defined as those yielding a ΔOD_620_ (t = 6 h − t = 0 h) ≤ 10% of the growth control [10]. To determine biofilm eradication, 10 µL from wells showing no visible growth were subcultured onto nutrient agar.

### 2.11. Statistics

All experiments were performed in triplicate, in three independent experimental sets. Data were expressed as mean ± SD. The results were evaluated by analysis of variance (ANOVA) using GraphPad Prism 8 computer software. In all analyses, p-values ≤ 5 were considered statistically significant.

## 3. Results

### 3.1. MIC and MBC assays

The MIC values of phendione, Ag‑phendione, Cu‑phendione, and the simple metal salts for the 35 EAEC clinical isolates are summarized in Table 2. Among the planktonic cells, the silver salt exhibited low antimicrobial activity (GM-MIC = 73.6 µM), whereas the copper salt was inactive under the conditions employed in the present study. The ligand phendione and its metal complexes demonstrated strong antimicrobial activity, with Cu‑phendione showing the greatest potency (GM-MIC = 4.03 µM), followed by Ag‑phendione (GM-MIC = 7.11 µM) and phendione (GM-MIC = 17.78 µM). The MBCs of all test compounds were also determined, with Cu‑phendione being the most effective (GM-MBC = 4.94 µM) (Table 2). For comparison, the susceptibility of *E. coli* reference strains to the phendione-derived compounds was also evaluated. All compounds inhibited the growth of these strains at low concentrations, with Cu-phendione exhibiting the strongest antimicrobial activity. Specifically, for ATCC 25922, MICs were 3.24 µM (Cu‑phendione), 9.96 µM (Ag‑phendione), and 14.88 µM (phendione); for ATCC 2469, respective MICs were 6.48, 9.96, and 14.88 µM; and for EAEC 042, respective MICs were 3.24, 9.96, and 14.88 µM. Interestingly, the MIC and MBC values determined for the reference strains were identical, indicating that the concentrations required for growth inhibition were the same as those needed to achieve bactericidal activity.

**Table 2.** MIC and MBC values of phendione, Cu‑phendione, Ag‑phendione, and simple salts against planktonic EAEC clinical isolates.

Compounds	MIC range, mg/L (µM)	MIC_50_, mg/L (µM)	MIC_100_, mg/L (µM)	GM-MIC, mg/L (µM)	MBC range, mg/L (µM)	GM-MBC, mg/L (µM)
Phendione	3.125–6.25 (14.88–29.74)	3.125 (14.88)	6.25 (29.74)	3.73 (17.78)	3.125–6.25 (14.88–29.74)	4.21 (20.02)
Ag-phendione	3.125–6.25 (4.98–9.96)	3.125 (4.98)	6.25 (9.96)	4.46 (7.11)	3.125–6.25 (4.98–9.96)	5.0 (7.97)
Cu-phendione	3.125–6.25 (3.24–6.48)	3.125 (3.24)	6.25 (6.48)	3.84 (4.03)	3.125–6.25 (3.24–6.48)	4.76 (4.94)
AgClO_4_	12.5 (73.6)	12.5 (73.6)	12.5 (73.6)	12.5 (73.6)	12.5 (73.6)	12.5 (73.6)
Cu(ClO_4_)_2_.6H_2_O	>100.0 (>400.50)	>100.0 (>400.50)	>100.0 (>400.50)	>100.0 (>400.50)	>100.0 (>400.50)	>100.0 (>400.50)

Note: GM-MIC: Geometric mean MIC; GM-MBC: Geometric mean MBC; ND: Not determined; MIC_50_ and MIC_100_: Minimum concentrations of test compounds required to inhibit 50% and 100% of the bacterial isolates, respectively.

### 3.2. Checkerboard assay

Based on the premise that the test compounds were capable of inhibiting the growth of EAEC clinical isolates resistant to conventional antimicrobials, we proceeded to employ the checkerboard method in order to further evaluate their potential synergistic or additive effects when combined with therapeutic antimicrobials. In this context, eight drug combinations were evaluated, consisting of ampicillin (AMP) or tetracycline (TET) in association with either Cu-phendione or Ag-phendione complexes, tested against eight AMP/TET-resistant isolates. The majority of the drug combinations (28/32; 87.5%) exhibited an additive effect (0.5 < FICI < 2.0). Synergistic interactions were observed in three cases: One isolate exposed to TET + Cu-phendione, one isolate exposed to AMP + Ag-phendione, and another isolate exposed to TET + Ag-phendione. One indifferent response was recorded for a single isolate treated with TET + Cu-phendione (Table 3). No antagonistic interactions were detected in any of the combinations tested.

**Table 3.** Checkerboard assay of Cu-phendione and Ag-phendione in combination with antimicrobials against tetracycline- and ampicillin-resistant EAEC clinical isolates.

Clinical isolates	MICs of single compounds (mg/L)				MICs of drug combinations (mg/L) (FICI value)			
	AMP	TET	Cu-phendione	Ag-phendione	AMP/Cu-phendione	AMP/Ag-phendione	TET/Cu-phendione	TET/Ag-phendione
146A	1024	128	6.25	6.25	64/3.125 (0.5625)	32/3.125 (0.53125)	64/6.25 (1.5)	32/3.125 (0.75)
Q287A	1024	64	3.125	3.125	256/1.5625 (0.75)	256/1.5625 (0.75)	4/1.5625 (0.5625)	16/0.78125 (0.50)
Q304A	1024	256	3.125	3.125	32/1.5625 (0.53125)	256/0.78125 (0.50)	64/0.78125 (0.5)	64/1.5625 (0.75)
Q320A	1024	64	6.25	6.25	256/3.125 (0.75)	128/3.125 (0.625)	64/6.25 (2.0)	32/3.125 (1.0)
Q323A	1024	128	6.25	6.25	32/3.125 (0.53125)	64/3.125 (0.5625)	64/6.25 (1.5)	32/3.125 (0.75)
Q345F	1024	128	3.125	6.25	32/1.5625 (0.53125)	64/3.125 (0.5625)	64/3.125 (1.5)	32/3.125 (0.75)
Q434A	1024	128	3.125	6.25	128/1.5625 (0.625)	64/3.125 (0.5625)	32/0.78125 (0.50)	32/3.125 (0.75)
Q436B	1024	128	3.125	6.25	128/1.5625 (0.625)	64/3.125 (0.5625)	32/1.5625 (0.75)	64/6.25 (1.5)

Note: AMP: Ampicillin; TET: Tetracycline; FICI: Fractional inhibitory concentration index; ND: Non-determined.

### 3.3. Time-kill assay

**Figure 1.**
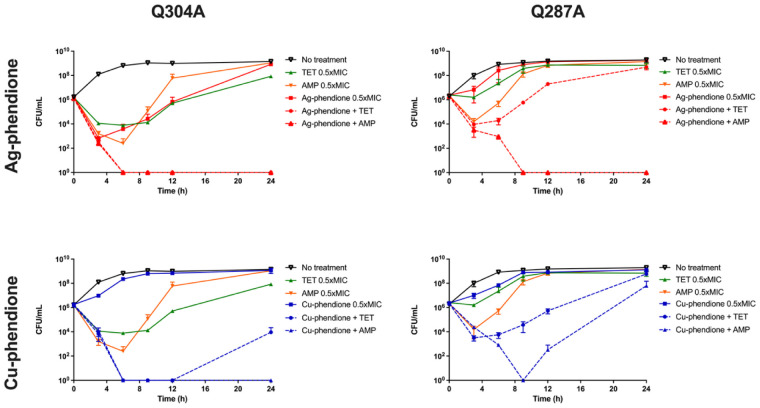
Time-kill curves of two EAEC clinical isolates treated with combinations of Cu‑phendione or Ag‑phendione and the antibiotics tetracycline (TET) or ampicillin (AMP) at 0.5× MIC of each agent.

The time-kill curves for the combinations of test compounds with therapeutic antimicrobials are presented in Figure 1. For the Q304A isolate, the combination of AMP or TET with either Cu-phendione or Ag-phendione produced a synergistic effect, with a reduction of >2 log_10_ compared to the most active single agent after 6 h of treatment, leading to undetectable CFU levels. After 24 h, no bacterial regrowth was observed with AMP + Cu-phendione, AMP + Ag-phendione, and TET + Ag-phendione, confirming the bactericidal activity of these combinations (reduction >3 log_10_ relative to the initial inoculum). In contrast, treatment with TET + Cu-phendione showed regrowth after 24 h, indicating incomplete eradication; however, the synergistic effect was still maintained (Figure 1).

For the Q287A isolate, TET + Cu-phendione, and TET + Ag-phendione exhibited a synergistic effect between 3 and 12 h and between 3 and 9 h of treatment, respectively; however, bacterial growth resumed in both combinations thereafter. The combination AMP + Cu-phendione showed synergism with bactericidal activity between 6 and 12 h, but regrowth occurred at 24 h (Figure 1). In contrast, AMP + Ag-phendione demonstrated both synergistic and bactericidal effects from 6 h onward, with CFU counts becoming undetectable by 9 h and remaining so through 24 h. Consequently, AMP + Ag-phendione was the most effective combination in both strains, exhibiting sustained synergism, bactericidal activity, and complete eradication of viable cells over 24 h.

### 3.4. MBIC and MBEC assays

MBIC values for the 35 EAEC clinical isolates treated with phendione, Ag-phendione, Cu-phendione, and the simple salts are summarized in Table 4. For biofilm-forming cells, the silver salt displayed low activity, with a high MBIC value (358.6 µM), whereas the copper salt showed no activity even at the highest concentration tested (MBIC > 801.0 µM). In contrast, phendione and its metal complexes were active against biofilm-growing cells, although higher concentrations were required compared to those effective against planktonic cells. Among them, Cu-phendione exhibited the greatest activity (GM-MBIC = 14.61 µM), followed by Ag-phendione (GM-MBIC = 24.69 µM) and phendione (GM-MBIC = 67.08 µM). MBEC values were also determined: The copper complex was the most effective in eradicating biofilms, achieving complete eradication in 24 isolates (68.6%). By comparison, phendione and Ag-phendione eradicated 6 (17.1%) and 11 (31.4%) isolates, respectively, at the concentrations tested.

**Table 4.** Effects of phendione, Cu-phendione, Ag-phendione, and simple salts on biofilm inhibition and eradication in EAEC clinical isolates.

Compounds	MBIC range mg/L (µM)	MBIC_50_ mg/L (µM)	MBIC_100_ mg/L (µM)	GM-MBIC mg/L (µM)	MBEC range mg/L (µM)
Phendione	6.25–25.0 (29.74–118.94)	12.5 (59.47)	25.0 (118.94)	14.1 (67.08)	12.5–>25.0 (59.47–>118.94)
Ag-phendione	12.5–50.0 (19.91–79.65)	12.5 (19.91)	25.0 (39.82)	15.5 (24.69)	12.5–>50.0 (19.91–>79.65)
Cu-phendione	6.25–25.0 (6.47–25.91)	12.5 (12.95)	25.0 (25.91)	14.1 (14.61)	6.25–>50.0 (6.47–>51.81)
AgClO_4_	25.0–100.0 (147.2–588.8)	50.0 (294.4)	100.0 (588.8)	60.9 (358.6)	>200.0 (1177.6)
Cu(ClO_4_)_2_.6H_2_O	>200.0 (>801.0)	>200.0 (>801.0)	>200.0 (>801.0)	>200.0 (>801.0)	>200.0 (>801.0)

Note: GM-MBIC: Geometric mean MBIC; MBIC_50_ and MBIC_100_: Minimum concentrations of test compounds required to inhibit 50% and 100% of the clinical isolates in biofilm, respectively.

### 3.5. Effects of test compounds on the disruption of mature biofilm

**Figure 2.**
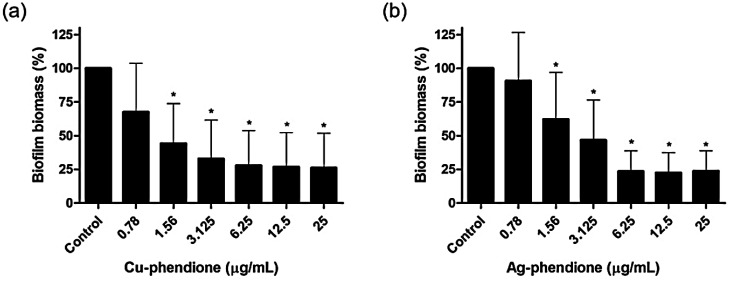
Percentage of EAEC biofilm biomass after treatment with Cu‑phendione (a) and Ag‑phendione (b) at concentrations ranging from 0.78 to 25 µg/mL. Columns represent the mean ± standard deviation of 35 EAEC clinical isolates tested at each concentration. Symbols indicate a significant difference compared to untreated biofilms (P < 0.05; one-way ANOVA, Dunnett's multiple comparison test).

The ability of Cu-phendione and Ag-phendione to disrupt mature biofilms formed by EAEC clinical isolates was thoroughly investigated. Both compounds exhibited a strong antibiofilm effect, reducing the established biomass by up to 75% (Figure 2). Notably, Cu-phendione promoted a significant reduction in biofilm biomass even at the lowest concentration tested (0.78 µg/mL), with no further enhancement observed between 1.56 and 25 µg/mL, suggesting a plateau effect. In contrast, Ag-phendione showed a concentration-dependent activity, with significant reductions detected from 1.56 µg/mL onward, reaching a maximum effect at 6.25 µg/mL, beyond which no additional reduction was observed. The calculated half-maximal inhibitory concentrations (IC_50_) confirmed the higher potency of Cu-phendione, with an IC_50_ value of 1.81 µg/mL (1.87 µM), compared to 3.03 µg/mL (4.83 µM) for Ag-phendione.

### 3.6. Ultrastructural images of biofilms

**Figure 3.**
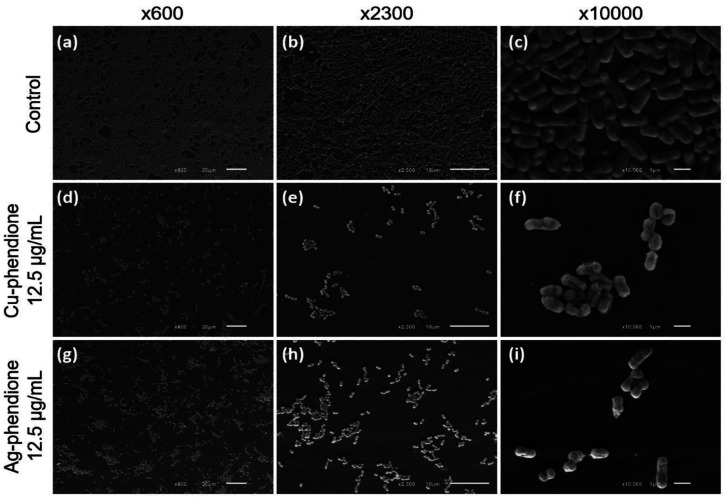
SEM images of EAEC biofilms: (a–c) untreated control; (d–f) after 24 h exposure to the MBIC of Cu‑phendione (12.5 µg/mL); (g–i) after 24 h exposure to the MBIC of Ag‑phendione (12.5 µg/mL). Both treatments significantly reduced cell size compared to the untreated control (P < 0.05). Cell sizes ranged from 0.64 to 1.65 µm (mean 1.17 ± 0.22 µm) in untreated biofilms, from 0.53 to 1.45 µm (mean 0.89 ± 0.24 µm) in Cu‑phendione-treated biofilms, and from 0.42 to 1.16 µm (mean 0.76 ± 0.16 µm) in Ag‑phendione-treated biofilms.

Scanning electron microscopy (SEM) was employed to further visualize the effects of Cu-phendione and Ag-phendione on EAEC biofilm architecture (Figure 3). For this set of experiments, the clinical isolate Q255D was selected due to its ability to form a robust biofilm [10]. In untreated controls, the biofilm exhibited a characteristic dense and compact three-dimensional architecture, with abundant bacterial aggregates. In contrast, treatment with the test complexes markedly altered this organization. Both complexes promoted a visible reduction in the number of adherent cells; however, the effect was more pronounced with Cu-phendione, where only sparse and scattered bacterial clusters remained attached to the abiotic surface. This loss of cohesion and spatial organization is consistent with the disarticulation of the mature biofilm structure, in sharp contrast to the tightly packed communities observed in untreated samples (Figure 3).

### 3.7. Effects of test compounds on biofilm formation kinetics

**Figure 4.**
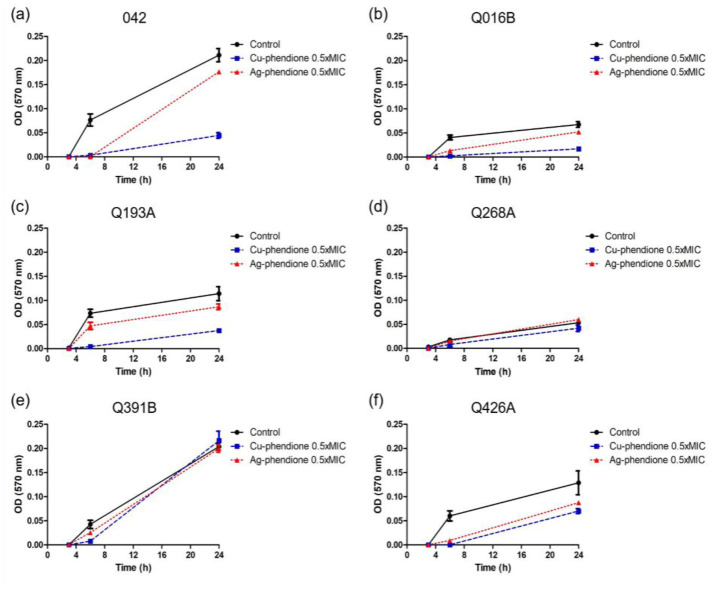
Representative biofilm formation kinetics for EAEC clinical isolates and the EAEC 042 prototype strain. Strains were incubated without compounds (black lines) or in the presence of Cu‑phendione (blue lines) and Ag‑phendione (red lines) at 0.5× MIC.

The inhibitory activity of the test complexes against EAEC biofilm formation was investigated at multiple time points in five clinical isolates, as well as in the prototype strain EAEC 042, using subinhibitory concentrations (0.5× MIC) of each compound (Figure 4). The results demonstrated that Cu-phendione exhibited the strongest inhibitory effect on biofilm formation, particularly at 6 h of incubation, achieving biomass reductions ranging from 55% to 100%. Moreover, Cu-phendione effectively prevented the full establishment of biofilms after 24 h in all isolates tested, with the exception of one isolate. Ag-phendione also reduced biofilm biomass after 6 h of incubation, with inhibition levels varying between 15% and 100%. However, in contrast to Cu-phendione, biofilm reestablishment was more evident after 24 h of incubation in the presence of Ag-phendione (Table 5).

**Table 5.** Reduction of biofilm biomass in EAEC clinical isolates and the reference strain 042 after exposure to 0.5× MIC of Cu-phendione and Ag-phendione for 6 and 24 h.

Bacterial strains	Biofilm reduction (%)			
	Cu-phendione		Ag-phendione	
	6 h	24 h	6 h	24 h
EAEC 042	95.63	78.99	100.00	16.43
Q016B	94.24	74.75	67.08	22.77
Q193A	94.55	67.25	35.91	23.98
Q268A	54.72	21.88	15.09	0
Q391B	82.03	0	40.63	1.96
Q426A	100.00	45.60	85.00	32.12

### 3.8. Effects of test compounds combined with therapeutic antimicrobials on biofilm inhibition and eradication

To investigate whether the association of the test compounds with conventional antimicrobials could lower the concentrations required to inhibit or even eradicate EAEC biofilms, we evaluated combinations of Cu-phendione and Ag-phendione with antimicrobials from different classes against four clinical isolates (Table 6). For this assay, antimicrobials were selected based on the susceptibility profile of the isolates, including drugs to which they were susceptible (cefoxitin, tobramycin, and ciprofloxacin) as well as those to which resistance was observed (ampicillin and tetracycline).

The combinations with Cu-phendione revealed a particularly strong synergistic effect. When associated with cefoxitin, the MBIC of cefoxitin decreased by up to 8-fold. Even more striking, combinations with tobramycin, ciprofloxacin, and tetracycline produced a 16-fold reduction in MBIC. The most remarkable result was observed with ampicillin: the Cu-phendione + ampicillin combination reduced the MBIC of ampicillin by up to 128-fold in the Q158A strain. Importantly, in isolates 139D, Q255D, and Q158A, which were initially resistant to ampicillin, the MBIC values were reduced to within the susceptibility range when the drug was combined with Cu-phendione.

Ag-phendione also enhanced antimicrobial efficacy, though to a lesser extent than Cu-phendione. Its combinations with cefoxitin, ciprofloxacin, and ampicillin reduced the MBIC of these antimicrobials by up to 4-fold. When combined with tetracycline, Ag-phendione produced up to a 16-fold reduction, while its association with tobramycin resulted in a 2-fold reduction.

Complete biofilm eradication was achieved in all tested strains when Cu-phendione was combined with cefoxitin, tobramycin, tetracycline, or ciprofloxacin, whereas its combination with ampicillin resulted in eradication in 50% of the strains. In contrast, Ag-phendione promoted complete biofilm eradication in all strains when combined with tobramycin or ciprofloxacin, and in 50% of the strains when combined with tetracycline. Notably, no eradication was observed for the combination of Ag-phendione with ampicillin.

**Table 6.** Effects of Cu-phendione and Ag-phendione in combination with therapeutic antimicrobials on EAEC biofilm inhibition and eradication.

Drug combinations	Bacterial isolates	MBIC			MBEC combination (A + B mg/L)
		Complexes (mg/L)	Antimicrobial (mg/L)	Combination (A + B mg/L)	
Cu-phendione (A) + Cefoxitin (B)	Q015B	12.5	4.0	6.25 + 2.0	12.5 + 8.0
	Q016B	12.5	4.0	6.25 + 0.5	1.56 + 8.0
	Q028B	12.5	8.0	6.25 + 4.0	12.5 + 16.0
	Q488B	12.5	4.0	12.5 + 4.0	25.0 + 8.0
Cu-phendione (A) + Tobramycin (B)	Q015B	12.5	2.0	6.25 + 0.125	1.56 + 2.0
	Q016B	12.5	4.0	6.25 + 1.0	12.5 + 2.0
	Q028B	12.5	2.0	12.5 + 2.0	1.56 + 2.0
	Q488B	12.5	2.0	1.56 + 1.0	12.5 + 0.5
Cu-phendione (A) + Tetracycline (B)	6A	25.0	128.0	12.5 + 8.0	25.0 + 64.0
	Q165A	12.5	128.0	1.56 + 64.0	25.0 + 32.0
	Q300A	12.5	128.0	12.5 + 128.0	6.25 + 256.0
	Q255D	25.0	128.0	12.5 + 32.0	25.0 + 64.0
Cu-phendione (A) + Ampicillin (B)	139D	12.5	16.0	1.56 + 2.0	NE
	Q255D	12.5	16.0	3.125 + 4.0	6.25 + 16.0
	Q158A	6.25	128.0	3.125 + 1.0	NE
	Q426A	12.5	32.0	12.5 + 32.0	6.25 + 128.0
Cu-phendione (A) + Ciprofloxacin (B)	Q015B	12.5	0.015	3.125 + 0.0075	12.5 + 0.0009
	Q016B	12.5	0.015	6.25 + 0.0009	6.25 + 0.0009
	Q028B	12.5	0.0075	12.5 + 0.0075	12.5 + 0.0009
	Q488B	12.5	0.0075	6.25 + 0.00375	3.125 + 0.0075
Ag-phendione (A) + Cefoxitin (B)	Q015B	12.5	4.0	3.125 + 2.0	NE
	Q016B	12.5	8.0	1.56 + 2.0	NE
	Q028B	25.0	8.0	6.25 + 4.0	NE
	Q488B	12.5	4.0	6.25 + 1.0	12.5 + 0.5
Ag-phendione (A) + Tobramycin (B)	Q015B	12.5	2.0	1.56 + 1.0	3.125 + 2.0
	Q016B	12.5	2.0	1.56 + 1.0	12.5 + 2.0
	Q028B	25.0	4.0	3.125 + 2.0	25.0 + 2.0
	Q488B	12.5	1.0	3.125 + 0.5	3.125 + 1.0
Ag-phendione (A) + Tetracycline (B)	6A	25.0	128.0	12.5 + 8.0	12.5 + 128.0
	Q165A	25.0	128.0	12.5 + 32.0	NE
	Q300A	12.5	128.0	12.5 + 128.0	25.0 + 128.0
	Q255D	12.5	128.0	1.56 + 8.0	NE
Ag-phendione (A) + Ampicillin (B)	139D	12.5	16.0	12.5 + 16.0	NE
	Q255D	12.5	16.0	3.125 + 4.0	NE
	Q158A	6.25	64.0	1.56 + 32.0	NE
	Q426A	25.0	64.0	1.56 + 32.0	NE
Ag-phendione (A) + Ciprofloxacin (B)	Q015B	12.5	0.015	12.5 + 0.015	3.125 + 0.015
	Q016B	12.5	0.03	1.56 + 0.015	6.25 + 0.0009
	Q028B	12.5	0.0075	12.5 + 0.0075	12.5 + 0.0009
	Q488B	12.5	0.0075	6.25 + 0.001875	3.125 + 0.0075

Note: MBIC: Minimum biofilm inhibitory concentration; MBEC: Minimum biofilm eradication concentration; NE: No eradication.

## 4. Discussion

The identification and susceptibility profiling of EAEC, an important bacterial enteropathogen, remain largely restricted to reference and research laboratories, leading to predominantly empirical therapeutic approaches. Alarmingly, isolates from diverse geographic regions have shown high resistance rates to the antimicrobials most frequently employed in empirical treatment [7]–[9]. This resistance profile becomes even more concerning when considering the biofilm mode of growth, a key feature of EAEC pathogenesis that contributes to chronic and recurrent infections [31]. In a recent study, we demonstrated that, among nine antimicrobials representing distinct therapeutic classes tested against biofilm-associated EAEC cells, only ciprofloxacin, tobramycin, and cefoxitin retained activity, whereas all others proved completely ineffective despite susceptibility in the planktonic state [10]. These findings highlight the urgent need for novel therapeutic strategies capable of targeting both planktonic and biofilm-associated forms of this ubiquitous pathogen. In this context, our results provide evidence that phendione-based complexes represent a promising alternative, either as standalone agents or in combination with conventional antimicrobials, particularly owing to their capacity to eradicate established biofilms.

Overall, phendione and its metal-based derivatives demonstrated pronounced inhibitory activity against diverse clinical isolates of EAEC exhibiting distinct susceptibility profiles to clinically employed antimicrobials. Importantly, these compounds were effective in targeting both planktonic populations and biofilm-associated cells, highlighting their potential as versatile agents capable of overcoming resistance mechanisms commonly observed in this pathogen. When planktonic growth was assessed by GM-MIC, Cu-phendione displayed the greatest potency, followed by Ag-phendione, whereas the free ligand (phendione) showed comparatively lower activity. This potency hierarchy is consistent with previous findings reported for other Gram-negative pathogens [11]–[13], underscoring the broad-spectrum relevance of these complexes. Notably, in addition to their activity against EAEC, the complexes also inhibited the planktonic growth of a carbapenem-resistant *E. coli* ATCC 2469 reference strain, corroborating earlier reports of their effectiveness against multidrug-resistant *P. aeruginosa*, *A. baumannii*, and *K. pneumoniae* [11]–[13].

With respect to the simple salts tested, Cu(ClO_4_)_2_.6H_2_O showed no detectable activity against either planktonic or biofilm-associated cells. In contrast, AgClO_4_ inhibited bacterial growth, but only at relatively high concentrations; an outcome consistent with the long-recognized antimicrobial properties of silver [32]. Notably, when comparing the activity of AgClO_4_ with that of the Ag-phendione complex, we observed substantial enhancements, with MIC and MBIC values reduced by 10.4- and 14.4-fold, respectively. These findings indicate that the antimicrobial effects cannot be attributed solely to the metal ion, but rather to the synergistic contribution of the metal–ligand complex. Furthermore, metal derivatives consistently outperformed the free phendione ligand, reinforcing the notion that complexation enhances biological activity, likely by increasing molecular lipophilicity and, consequently, facilitating cellular uptake and access to intracellular targets [33].

In recent years, a variety of therapeutic strategies have been investigated to combat infections caused by resistant bacteria, including the use of antimicrobial combinations and the development of adjuvant compounds capable of lowering the inhibitory concentrations of conventional drugs to clinically relevant levels, thereby restoring their effectiveness [33]–[35]. In this context, Peregrino et al. [13] demonstrated a remarkable resensitization of KPC-producing *K. pneumoniae* when phendione-derived complexes were combined with carbapenems. Seeking to explore a similar effect in EAEC, we evaluated, using the checkerboard method, the interaction of phendione-based compounds with ampicillin and tetracycline, two antimicrobials to which our clinical isolates exhibited the highest resistance rates. However, no resensitization was detected, likely due to the extremely elevated MIC values observed for these drugs (1024 µg/mL for ampicillin and 32–256 µg/mL for tetracycline). Interestingly, when the combinations were assessed using a time-kill assay, considered the gold standard for evaluating drug interactions, synergistic effects emerged. At 0.5× MIC of each agent, both ampicillin and tetracycline displayed synergy with the complexes. Notably, Ag-phendione in combination with ampicillin yielded the most striking outcome; in addition to exhibiting synergism and bactericidal activity, it completely prevented bacterial regrowth for at least 24 h in all tested strains. These results parallel previous findings with *K. pneumoniae*, where the association of Ag-phendione and meropenem achieved comparable efficacy [13]. Taken together, these findings highlight the potential of achieving bacterial eradication through the use of subinhibitory concentrations of conventional antimicrobials when combined with phendione-derived complexes, thereby reinforcing the promise of combination therapy as an alternative strategy for treating resistant strains. In a similar vein, Morones-Ramirez et al. [32] demonstrated that silver can function as a potent adjuvant, enhancing the activity of a broad spectrum of antimicrobials against *E. coli* in diverse metabolic states, including persister cells, in both planktonic and biofilm-associated forms. This potentiating effect was attributed to silver's capacity to disrupt key bacterial cellular processes, which results in increased reactive oxygen species production and heightened membrane permeability in Gram-negative bacteria, ultimately facilitating antimicrobial penetration and activity.

All tested complexes exhibited antibiofilm activity; however, Cu-phendione stood out as the most potent, displaying lower MBIC values, higher rates of biofilm eradication, strong inhibition of biofilm formation, and effective disruption of mature biofilms, as evidenced by its lower IC₅₀ value. Complementary SEM analyses further confirmed these findings, revealing a markedly reduced number of adherent bacteria in biofilms treated with this compound. Consistent with our observations, Cu-phendione has also been reported to exhibit superior antibiofilm activity against *P. aeruginosa* and *A. baumannii* isolates when compared with other phendione-based derivatives, underscoring its therapeutic potential in infections where biofilm formation is a major virulence factor [11],[12]. In agreement with these data, Beeton et al. [36] demonstrated that copper complexes significantly reduce the biofilm biomass of methicillin-resistant *S*. *aureus* (MRSA), with effects surpassing those of vancomycin, a first-line antimicrobial for MRSA treatment. This enhanced activity was attributed to the ability of copper coordination complexes to cleave DNA. In a similar manner, Cu-phendione has been shown to induce oxidative DNA damage and fragmentation in *P. aeruginosa* [17], providing further mechanistic support for the role of copper-based compounds in targeting microbial DNA and compromising biofilm integrity.

The persistence of biofilm-associated infections is largely attributed to the presence of persister cells, which represent a transiently dormant subpopulation tolerant to antimicrobial treatment. This phenomenon is particularly evident with ciprofloxacin, an antibiotic that readily penetrates the extracellular matrix and effectively kills the majority of bacterial cells, yet simultaneously triggers stringent response pathways. As a result, a fraction of the population shifts into a persister phenotype, ultimately leading to therapeutic failure and incomplete biofilm eradication [37],[38]. Remarkably, the metal-based compounds tested in this study, most notably Cu-phendione, were more effective in eradicating EAEC biofilms than conventional antimicrobials previously evaluated, either as monotherapies or in combination regimens, including ciprofloxacin [10]. These results strongly suggest that such compounds may exert activity against persister cells, overcoming one of the most critical barriers in the treatment of biofilm-associated infections.

Evaluating the effect of the combined therapy on biofilm, we observed that combinations with Cu-phendione proved to be the most effective compound in reducing the MBIC of antimicrobials, achieving resensitization of two ampicillin-resistant strains and promoting biofilm eradication across all isolates when combined with nearly all antimicrobials tested. This enhanced effect may be attributed to its capacity to act on persister cells, a subpopulation typically unaffected by conventional antibiotics. Notably, eradication was observed even in combination with antimicrobials to which the strains were resistant, such as tetracycline and ampicillin; however, in these cases, concentrations above the established susceptibility breakpoints were required. These findings underscore the potential of combining antimicrobials with metal-phendione complexes as a promising therapeutic strategy. Since the two classes of agents target distinct cellular processes, their combined use enables a multifaceted attack on bacterial populations, thereby enhancing overall efficacy and reducing the likelihood of therapeutic failure [39].

## 5. Conclusions

In conclusion, the findings of the present study demonstrate that phendione-derived metal complexes exhibit potent antimicrobial activity against EAEC clinical isolates, effectively targeting both planktonic and biofilm-associated cells, and achieving biofilm eradication in the majority of isolates. These promising results, aligned with previous evidence, highlight the potential of such compounds as valuable candidates for antimicrobial therapy, particularly in the context of drug-resistant infections. Nonetheless, further investigations addressing pharmacokinetics, in vivo efficacy, and underlying mechanisms of action are essential to advance their translational development and clinical applicability.

## Use of AI tools declaration

The authors declare they have not used Artificial Intelligence (AI) tools in the creation of this article.
